# Visuomotor processing is altered after peripheral nerve damage in neuralgic amyotrophy

**DOI:** 10.1093/braincomms/fcac034

**Published:** 2022-02-16

**Authors:** Renee Lustenhouwer, Ian G. M. Cameron, Elze Wolfs, Nens van Alfen, Ivan Toni, Alexander C. H. Geurts, Baziel G. M. van Engelen, Jan T. Groothuis, Rick C. Helmich

**Affiliations:** 1 Department of Rehabilitation, Radboud University Medical Center, Donders Institute for Brain, Cognition and Behaviour, Nijmegen, The Netherlands; 2 Donders Centre for Cognitive Neuroimaging, Donders Institute for Brain, Cognition and Behaviour, Radboud University, Nijmegen, The Netherlands; 3 Donders Centre for Neuroscience, Donders Institute for Brain, Cognition and Behaviour, Radboud University, Nijmegen, The Netherlands; 4 Faculty of Electrical Engineering, Mathematics and Computer Science, University of Twente, PO BOX 217, 7500 AE Enschede, The Netherlands; 5 Department of Experimental Psychology, Helmholtz Institute, Utrecht University, Heidelberglaan 1, 3584 CS Utrecht, The Netherlands; 6 Department of Neurology, Radboud University Medical Center, Donders Institute for Brain, Cognition and Behaviour, Nijmegen, The Netherlands

**Keywords:** fMRI, mental hand rotation, motor imagery, maladaptive plasticity, sensorimotor system

## Abstract

Neuralgic amyotrophy is a common peripheral nerve disorder caused by autoimmune inflammation of the brachial plexus, clinically characterized by acute pain and weakness of the shoulder muscles, followed by motor impairment. Despite recovery of the peripheral nerves, patients often have residual motor dysfunction of the upper extremity, leading to persistent pain related to altered biomechanics of the shoulder region. Building on clinical signs that suggest a role for cerebral mechanisms in these residual complaints, here we show and characterize cerebral alterations following neuralgic amyotrophy. Neuralgic amyotrophy patients often develop alternative motor strategies, which suggests that (mal)adaptations may occur in somatomotor and/or visuomotor brain areas. Here, we tested where changes in cerebral sensorimotor representations occur in neuralgic amyotrophy, while controlling for altered motor execution due to peripheral neuropathy. We additionally explore the relation between potential cerebral alterations in neuralgic amyotrophy and clinical symptoms. During functional MRI scanning, 39 neuralgic amyotrophy patients with persistent, lateralized symptoms in the right upper extremity and 23 matched healthy participants solved a hand laterality judgement task that can activate sensorimotor representations of the upper extremity, across somatomotor and visuomotor brain areas. Behavioural and cerebral responses confirmed the involvement of embodied, sensorimotor processes across groups. Compared with healthy participants, neuralgic amyotrophy patients were slower in hand laterality judgement and had decreased cerebral activity specific to their affected limb in two higher-order visual brain regions: the right extrastriate cortex and the parieto-occipital sulcus. Exploratory analyses revealed that across patients, extrastriate activity specific to the affected limb decreased as persistent pain increased, and affected limb-related parieto-occipital activity decreased as imagery performance of the affected limb became slower. These findings suggest that maladaptive cerebral plasticity in visuomotor areas involved in sensorimotor integration plays a role in residual motor dysfunction and subsequent persistent pain in neuralgic amyotrophy. Rehabilitation interventions that apply visuomotor strategies to improve sensorimotor integration may help to treat neuralgic amyotrophy patients.

## Teaser

Peripheral nerve disorders may entail cerebral adaptations that contribute to clinical symptoms. In neuralgic amyotrophy, using functional MRI Lustenhouwer *et al*. observed disease-specific cerebral dysfunction in visuomotor brain areas. This identifies visuomotor integration as a possible target for treatment.

## Graphical Abstract

**Graphical Abstract:**
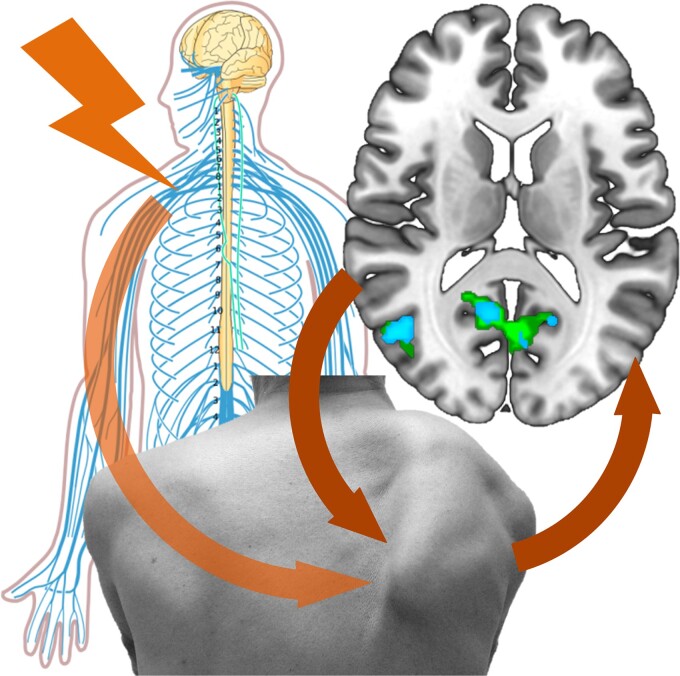

## Introduction

When elements of the sensorimotor system are damaged, motor function can be regained through plastic adaptations within the nervous system.^1,2^ However, this reorganization is not always clinically beneficial, and may even contribute to impaired motor function, in which case it is considered maladaptive.^1,3^ Maladaptive neuroplasticity has been linked to motor dysfunction in several central and peripheral nervous system disorders.^4–9^ A striking clinical example of the latter is obstetric brachial plexus palsy, which is associated with developmental apraxia and persistent clinical motor dysfunction despite peripheral reinnervation.^10^ Persistent motor dysfunctions following recovery from peripheral nerve damage offer a well-defined test case for understanding mechanisms of central neuroplasticity.

Here, we study cerebral changes related to persistent motor dysfunction and subsequent pain in neuralgic amyotrophy (NA), a common (incidence of 1/1000) and disabling peripheral nerve disorder that involves acute autoimmune inflammation of the brachial plexus.^11–13^ NA is typically asymmetric and most often involves one upper extremity.^14^ The characteristic paresis of muscles that are innervated by damaged nerves leads to motor impairment of the affected limb, most notably the shoulder. Many patients subsequently develop alternative movement patterns that can be beneficial for compensation at first, but may lead to long-term motor dysfunction and persistent pain in the long run. These secondary impairments are related to overuse of and strain of compensating muscles and shoulder impingement due to altered scapular biomechanics.^12,13,15^ Several clinical signs connect these residual complaints to maladaptive cerebral changes. First, patients often do not regain motor function, despite reinnervation of the affected muscles and return of muscle strength.^12–15^ Second, some NA patients develop abnormal and involuntary movements that resemble dystonia, a symptom which is often associated with cerebral abnormalities.^16^ Third, patients can regain normal motor function through specialized rehabilitation that focuses on relearning correct movement patterns and postures, even years after an onset.^17^ Finally, we have recently shown in a separate behavioural study that NA patients have behavioural deficits during motor imagery of the affected limb, as evoked by a hand laterality judgement task, suggesting that NA patients have altered sensorimotor representations related to their affected limb.^18^ However, it remains unclear whether these alterations arise from changes in somatomotor processes, visuomotor processes or both.

We address this issue in an independent sample of NA patients with task-based functional MRI (fMRI) during performance of the hand laterality judgement task, which involves both somatomotor and visuomotor processes, and can activate sensorimotor representations of the upper limb.^19–23^ In this task, participants have to judge the laterality (left or right) of hand stimuli. It is believed that subjects solve this task by mentally rotating their own body part to match the stimulus, a process involving ‘motor imagery’: mental simulation of movement, without overt motor expression.^24,25^ This validated task involves similar cerebral processes as motor planning,^19,20,26^ without interference of disease-related abnormalities in motor execution or associated afferent feedback.^25,27,28^ The fact that subjects incorporate their own body posture when performing the task, suggests that this task has embodied components.^21–23^ This task typically engages a fronto-parieto-occipital network that includes key motor regions such as premotor cortex and supplementary motor area, as well as areas in the posterior parietal cortex along the dorsal visual stream^21,22,26,27,29–31^ The hand laterality judgement task has been shown to be sensitive to altered cerebral processes in other asymmetric central and peripheral neurological disorders of the upper limb, as evidenced by behavioural deficits found in focal hand dystonia,^28^ carpal tunnel syndrome,^32^ traumatic brachial plexus injury^33^ and during brachial plexus anaesthesia.^34^ Interestingly, fMRI studies in Parkinson’s disease have shown that the hand laterality judgement task can detect cerebral changes across the whole fronto-parietal-occipital network, including occipito-parietal regions outside the pathological substrate of this central nervous system disorder.^29,30^ Accordingly, here we use fMRI during performance of the hand laterality judgement task to characterize somatomotor and/or visuomotor cerebral alterations in NA. We additionally explore the relation between these potential cerebral alterations and clinical symptoms.

## Materials and methods

This is a sub-study of a randomized controlled trial (RCT), which investigates the effect of specialized rehabilitation on residual complaints in NA, in addition to the role that cerebral mechanisms may play in patients’ persistent motor problems. All MRI-compatible individuals participating in the RCT were included in the current sub-study. We used the fMRI and relevant clinical data collected during the pre-treatment baseline assessment to compare with the healthy participants. For an extensive description of the RCT see Lustenhouwer *et al*.^35^ The study was approved by the local medical ethical committee (Medical Ethical Committee region Arnhem-Nijmegen, CMO 2017-3740) and is registered at ClinicalTrials.gov (NCT03441347).

### Participants

Forty-seven patients with a right-sided NA of the brachial plexus were included as part of the RCT (see Table 1 for details). Due to the COVID-19 pandemic and subsequent national measures, inclusion was terminated short of the original goal of 50 patients. Twenty-five age- and sex-matched healthy participants additionally participated. All participants were ≥18 years of age and had right hand dominance (as evidenced by a score of >+40 on the Edinburgh Handedness Inventory^36^).

**Table 1:** Participant characteristics of the participants that were included in the final analyses

	Neuralgic amyotrophy patients	Healthy participants
Age (years)	43 ± 11	43 ± 9
Sex (male/female) (%)	23/16 (60/40%)	13/10 (57/43%)
Time since last attack	17 ± 34	—
(months)	8, 2–204	
median, min–max		
DASH score	39 ± 19	—
Pain (VAS)	30 ± 26	—
Serratus anterior strength (Newton)		
Left (unaffected/non-dominant) side	236.5 ± 6.3^a^	235.4 ± 6.3^b^
Right (affected/dominant) side	195.9 ± 9.9^a,c^	247.8 ± 7.1^b,c^

Mean ± SD are displayed for all measures, except for the serratus anterior strength which shows the mean ± SEM.

NA = neuralgic amyotrophy; DASH = disabilities of arm, shoulder and hand; VAS = visual analogue scale.

^a^ Significant difference between left and right serratus strength within NA patients (*P* < 0.001).

^b^ Significant difference between left and right serratus strength within healthy participants (*P* = 0.010).

^c^ Significant difference in right serratus strength between NA patients and healthy participants (*P* < 0.001).

NA patients who presented with clearly lateralized symptoms of the right upper extremity, exhibited explicit coordinative motor dysfunction (i.e. scapular dyskinesia), who were no longer in the acute inflammatory phase (i.e. >8 weeks after attack onset), had not yet received specialized rehabilitation care and had no relevant comorbidities, were recruited through the Neuromuscular Center of the Radboud university medical center. HPs without current or previous shoulder problems and other relevant comorbidities (e.g. neurological or muscular disorders) were recruited through the university’s subject database. See Lustenhouwer *et al*.^35^ for a detailed description of the recruitment procedures and in- and exclusion criteria.

Data from 39 NA patients and 23 healthy participants were analysed. We excluded three patients because of contraindications for MRI, two patients had bilateral NA, one patient and one healthy participant had a pre-existing condition missed at initial screening, one patient was excluded due to movement during the MRI scan (mean framewise displacement >0.5 mm) and one patient and one healthy participant due to high behavioural error rates (ERs) on the hand laterality judgement task [>group mean plus 3 standard deviations (SDs)].

### Sample size calculation

The RCT was powered to demonstrate clinical effects of a specialized rehabilitation programme on functional capability of the upper limb in NA patients.^35^ The current sample (39 NA patients, 23 healthy participants) suffices to replicate our previous behavioural finding of a significant interaction effect of GROUP × LATERALITY on ER on the same task in an independent sample of NA patients^18^ (with power at 0.90 and *α* at 0.05, the required sample size is 14).^37^ Previous fMRI studies using the same task have found differences in brain activity between patient populations and healthy participants with sample sizes similar to the current sample.^27,29^

### Procedures

All participants were briefed on the nature of the study and gave written informed consent prior to participation according to the Declaration of Helsinki. The study included multiple assessments (see Lustenhouwer *et al*.^35^). All participants started with the MRI session consisting of a structural scan, a resting-state fMRI scan (not included in this study) and the task-fMRI scan (described in detail below). Following the MRI session, we collected several objective and subjective clinical measures to quantify NA-related symptoms: the serratus anterior muscle strength on both the right (affected) and left (unaffected) side, the disabilities of the arm, shoulder and hand (DASH) questionnaire^38^ and pain. We estimated the maximal force exerted with the serratus anterior muscle, which is often affected in NA patients,^14^ with a manual digital dynamometer (MicroFET2®).^39^ The DASH is a validated questionnaire that measures the functional capability of the upper extremity. Scores range from 1 to 100 with higher scores reflecting more impairment. Patients were asked to indicate how much pain they currently experienced on a visual analogue scale (VAS), ranging from no pain (0) to unbearable pain (100).^40^

### Experimental design

**Figure 1:**
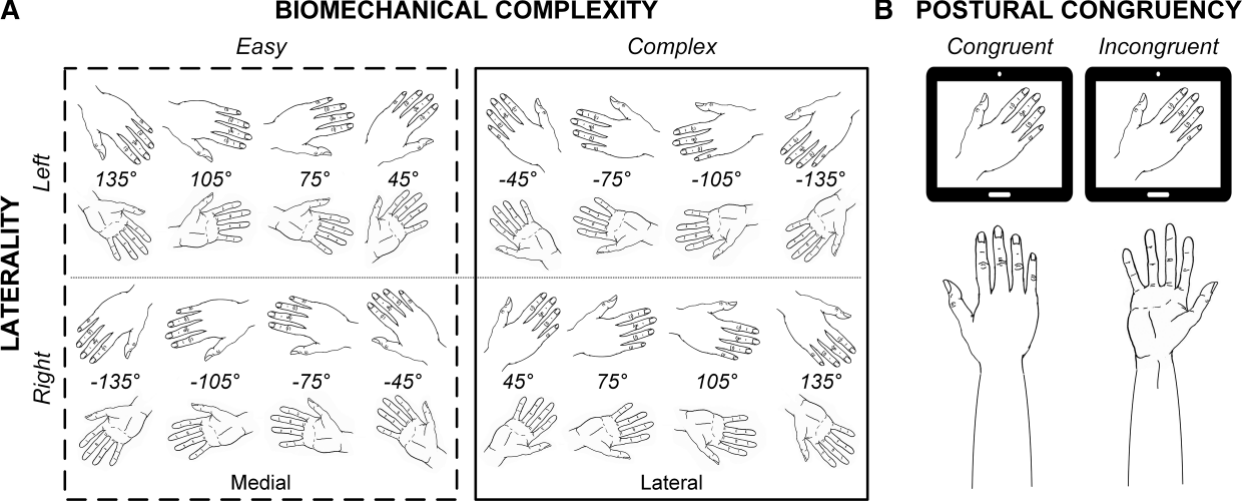
**Experimental design**. (**A**) Stimulus overview. Stimuli differed in laterality (left, right), view (dorsal, uneven rows and palmar, even rows) and were rotated from −135° to 135°, in 30° increments. Hands with a medial orientation were associated with biomechanically easy movement, whereas hands with a lateral orientation were associated with biomechanically complex movement. (**B**) Postural manipulation. Participants were instructed to assume a limb position (with the palm of their hand up or down) at the start of each block. The current position of their own limb could either be congruent with the view (dorsal or palmar) of the stimulus on the screen (see congruent example), or it could be incongruent (see incongruent example).

All individuals performed the hand laterality judgement task^25^ in the fMRI scanner. Participants were presented with white line drawings of hands on a black background (the stimulus). Their task was to judge whether the hand on display represented a left or a right hand. They were instructed to respond as quickly and accurately as possible and to use their own hands as reference (such that they could *imagine* making a limb movement to match the hand on the screen), but that they were not allowed to overtly move their own limbs (this was confirmed using EMG, see below). Participants could not rely on visual input, since they were not able to see their own hands. Stimuli varied in laterality (left or right), degree of rotation (rotated −135°, −105°, −75°, −45°, 45°, 75°, 105°, 135° from the upright position), and view (palmar or dorsal), amounting to 32 different stimuli (Fig. 1A). Different rotations and views were included as engagement of sensorimotor processes critically relies on these factors.^25,41^ Moreover, they enable the assessment of two factors of interest. First, BMC: stimuli either have a medial (i.e. hand rotated towards the body midsagittal plane) or a lateral orientation (i.e. hand rotated away from the body midsagittal plane). Medially oriented hands are in more comfortable positions, making medial trials biomechanically easy to perform, whereas lateral orientations are biomechanically complex. This results in laterally oriented stimuli eliciting longer reaction times (RTs) than medially oriented stimuli known as the ‘biomechanical complexity effect’.^19–21,24,25^ Second, the compatibility of the presented hand stimulus with the posture of the participant’s own hand. More specifically, the inclusion of the two different views (palm or back) enabled us to assess the effect of postural manipulation (Fig. 1B). With this manipulation, we could test whether participants incorporated their own body posture during the task. At the start of each block, participants were instructed to place their own limbs in one of four possible positions: both hands with palms facing up, both hands palms facing down, one hand palm up (left/right) and the other hand palm down (right/left). The view of the stimulus on the screen could either be congruent or incongruent with the current position of the corresponding limb. Typically, participants are slower when their corresponding limb is in an incongruent compared with a congruent position, which indicates that this task comprises embodied components.^21–23^

Each trial started with a white fixation cross in the centre of the screen, followed by the stimulus. The stimulus was presented until a response was registered, with a maximum of 4 s. Participants responded by pressing a foot button with the corresponding left or right great toe. The inter-trial interval varied randomly between 2000 and 3000 ms. The task consisted of 32 blocks of 8 trials (totalling 256 trials; duration 20–30 min). The trial order was pseudo-randomized, ensuring that the different trial types were spread evenly across blocks. We recorded RT and ER to evaluate behavioural performance. Throughout the task we additionally monitored muscle activity, using EMG over both thumbs (thenar eminence) to rule out that participants made hand movements during the trails. Alertness was monitored through an online eye monitor.

Participants first performed four blocks of eight practice trials at a desktop computer to familiarize themselves with the task. During scanning, the participants lay in a supine position on the scanner bed, with their head fixed in the MRI head coil, a piece of tape attached to their forehead to minimize movement^42^ and their extended arms resting on the scanner bed, support pillows or their thighs. The stimulus screen was visible through a mirror mounted to the head coil.

All images were acquired on a 3T Siemens PrismaFit scanner (Siemens Healthcare, Erlangen, Germany), equipped with a 32-channel head coil. A T_1_-weighted anatomical scan was acquired with a magnetization prepared rapid gradient echo (MPRAGE, TR = 2300 ms, TE = 3.03 ms, TI = 1100 ms, flip angle = 8°, voxel size = 1.0 × 1.0 × 1.0 mm, slices = 192, FOV = 256 mm, scanning time = 5:21 min). Functional scans were acquired during the task with a multiband six sequence (MB6, TR = 1000 ms, TE = 34 ms, acceleration factor = 6, flip angle = 60°, voxel size = 2.019 × 2.019 × 2.000 mm, slices = 72, FOV = 210 mm, scanning time = 20–30 min dependent on task performance).

### Preprocessing of neuroimaging data

Preprocessing was performed with FSL version 5.0.11 (FMRIB’s Software Library, Oxford, UK).^43^ We first removed non-brain structures from the structural image using the brain extraction tool (BET).^44^ Functional images were realigned with fMRI expert analysis tool (FEAT).^45^ FEAT additionally applied smoothing [full width at half maximum (FWHM) = 3 mm] and grand mean scaling and removed non-brain structures from the functional images, which were then registered to the structural image and standard MNI152 space using linear (FLIRT) and non-linear registration (FNIRT).^46–49^ Motion-related noise was removed using ICA-AROMA.^50^ We manually inspected and, if needed, reclassified^51^ the 100 independent components that ICA-AROMA generated per participant as noise or signal, after which we applied non-aggressive denoizing. Next, we performed nuisance regression on the denoized images, which included regressors of white matter and cerebrospinal fluid, as well as 24 motion parameters.^52^ After nuisance regression, we temporally high pass filtered the data at 0.01 Hz and applied additional smoothing with a 5.2 mm FWHM Gaussian kernel, amounting to a final smoothing of 6 mm FWHM. See the Supplementary materials for a more detailed description of all preprocessing steps.

### Behavioural analyses

Statistical testing was performed using the IBM SPSS statistics 25. Unless otherwise specified, statistical tests were two-tailed and alpha-level was set at *P* = 0.05. We made a comparison between NA patients and healthy participants using a chi square test for sex, and an independent samples *t*-test for age. Serratus anterior muscle strength of both limbs was compared with a 2-factor mixed ANOVA, which included repeated factor SIDE (left, right) and between-factor GROUP (NA, healthy). To evaluate task performance, we calculated median RTs (on correct trials) and ERs (i.e. number of incorrect trials divided by number of valid (correct + incorrect) trials) for all relevant conditions. Before statistical analyses, ER was normalized through an arcsine transformation.^53^

We tested for the effects of between-group factor GROUP (NA, healthy) and repeated factors BIOMECHANICAL COMPLEXITY (easy, complex) and LATERALITY (left, right) on median RT, and normalized ER with two separate 3-factor mixed ANOVAs. We additionally analysed the influence of between-group factor GROUP, and repeated factors POSTURE (congruent, incongruent) and LATERALITY on median RT and normalized ER with two additional 3-factor mixed ANOVAs. To correct for multiple testing (4 ANOVAs) we set alpha at *P* = 0.0125 for these analyses.

### Task-related cerebral activity

Image analyses were performed using the FSL version 5.0.11.^43^ On the first (subject-specific) level, we used a general linear model (GLM) to model blood-oxygen-level dependent (BOLD) activation per participant.^54,55^ Task design matrices and contrast images of parameter estimates of the BOLD signal were generated using FEAT.^45^ Our statistical model at the first level included the factors LATERALITY (left, right), BIOMECHANICAL COMPLEXITY (easy, complex) and POSTURE (congruent, incongruent). We additionally included interaction terms for LATERALITY × BIOMECHANICAL COMPLEXITY and LATERALITY × POSTURE, to investigate the limb-specific effect of movement complexity and somatosensory changes on activation, respectively. All regressors of interest included correct responses only, and events were time-locked to stimulus onset, with a duration corresponding to the median RT over all trials per participant, following the same approach as in previous work in healthy and clinical populations.^21,27,29,30,56^ The model additionally included three regressors of non-interest: between block hand re-positioning (7 s), incorrect trials and missed trials (with median RT durations). All regressors were convolved with a canonical hemodynamic response function (HRF) and its temporal derivative to model BOLD activation.^57^ FMRIB’s improved linear modelling (FILM) pre-whitening was performed before voxel-wise fitting of the GLM.^45^

Group analyses were done using FSL’s randomize tool^58^ to perform non-parametric threshold-free cluster enhancement (TFCE) based permutation testing (5000 permutations), correcting for multiple comparisons with a family wise error (FWE) of *P* < 0.05. For each contrast of interest, the GLM consisted of factor GROUP (NA or healthy), with the individual contrast of parameter estimate images from the first level analyses as input. Contrasts of interest were LATERALITY (left > right, right > left), BIOMECHANICAL COMPLEXITY (complex > easy) and POSTURE (incongruent > congruent), as well as their interaction terms (right_complex>easy_ > left_complex>easy,_ right_incongruent>congruent_ > left_incongruent>congruent_). We additionally looked for shared activity and general task effects by running a single group simple design for the contrasts relating to factors LATERALITY (left > right, right > left), BIOMECHANICAL COMPLEXITY (complex > easy, collapsed over LATERALITY and for left and right hands separately) and POSTURE (incongruent > congruent).

#### *Post hoc* functional connectivity analysis

As NA patients showed altered brain activity compared with healthy participants during mental rotation of right (affected) versus left (non-affected) hands in two brain regions (see Results), we performed *post hoc* seed-based functional connectivity analyses to explore the underlying networks. To this end, we extracted the mean BOLD signal within those regions (MNI coordinates peak voxel, right extrastriate cortex: [50 −66 12], 114 voxels; bilateral parieto-occipital sulcus: [10 −58 16], 429 voxels; see Table 2) and ran two separate GLMs with the timeseries of each of the regions as a single regressor. For each of the regions, we compared the resulting contrast of parameter estimate images between groups with a non-parametric two-sample *t*-test, using TFCE-based permutation testing (5000 permutations).^58^

**Table 2:** Group difference in motor imagery-related activity table containing information on significant clusters for the interactions between GROUP × LATERALITY × BIOMECHANICAL COMPLEXITY, and GROUP × LATERALITY for complex stimuli only

Anatomical region	Cluster probability (%) Juelich histological atlas		*P*-value (FWE-corrected)	Cluster size (voxels)	TFCE (peak voxel)	Stereotactic coordinates (MNI)		
						*x*	*y*	*z*
GROUP × LATERALITY × BMC: right_BMC_ > left_BMC_, healthy > NA								
R extrastriate cortex	R inferior parietal lobule area PGp R V5	18% 12%	0.011	114	13 544	50	−66	12
Bilateral parieto-occipital sulcus	L V2 BA18 R V2 BA18 L V1 BA17 R V1 BA17	10% 8% 5% 6%	0.018	429	12 679	10	−58	16
GROUP × LATERALITY(complex): right_complex_ > left_complex_, healthy > NA								
Bilateral parieto-occipital sulcus	L V2 BA18 R V2 BA18 L V1 BA17 R V1 BA17	7% 7% 7% 5%	0.004	1245	17 553	4	−74	24
R extrastriate cortex	R inferior parietal lobule area PGp R V5	21% 10%	0.027	82	13 167	50	−64	14
Bilateral superior parietal lobule	L 7A R 7A	5% 5%	0.041	21	12 098	0	−64	64

BMC = biomechanical complexity; left/right_BMC_ = biomechanical complexity effect for left/right hands; L = left; NA = neuralgic amyotrophy; R = right; FWE = familywise error; TFCE = threshold-free cluster enhancement.

#### Brain-behaviour-symptom correlations

In exploratory analyses, we correlated task-related cerebral activity in clusters showing significant effects involving group (NA, healthy) with relevant clinical measures (functional capability of the upper limb (DASH score), persistent pain (VAS-score) and relative serratus anterior muscle strength (i.e. affected minus unaffected muscle strength), and with behavioural performance measures (matching the cerebral effects and those showing significant group effects). We additionally explored correlations between the same behavioural and clinical measures.

## Data availability statement

The data are available through the corresponding author on reasonable request.

## Results

Table 1 shows the characteristics of participants included in data analysis. There were no significant group differences in age [*t*(60) = 0.04, *P* = 0.97], or sex [*χ*^2^(1) = 0.04, *P* = 0.85]. The analysis of serratus anterior strength yielded a significant GROUP × SIDE interaction effect [*F*(1,60) = 20.5, *P* < 0.001, part. *η*^2^ = 0.26]. NA patients exerted significantly less strength with their affected, right (dominant) compared with their left serratus anterior muscle [*F*(1,38) = 22.4, *P* < 0.001, part. *η*^2^ = 0.37], whereas healthy participants exerted significantly more strength with the serratus anterior muscle on their right (dominant), compared with their left side [*F*(1,22) = 7.9, *P* = 0.010, part. *η*^2^ = 0.26]. Moreover, NA patients exerted significantly less force with their right serratus anterior muscle than healthy participants [*F*(1,61) = 13.6, *P* < 0.001, part. *η*^2^ = 0.19]. This confirms that NA patients had lateralized symptoms of the right upper limb (Table 1).

### Behavioural results

#### Reaction times

**Figure 2:**
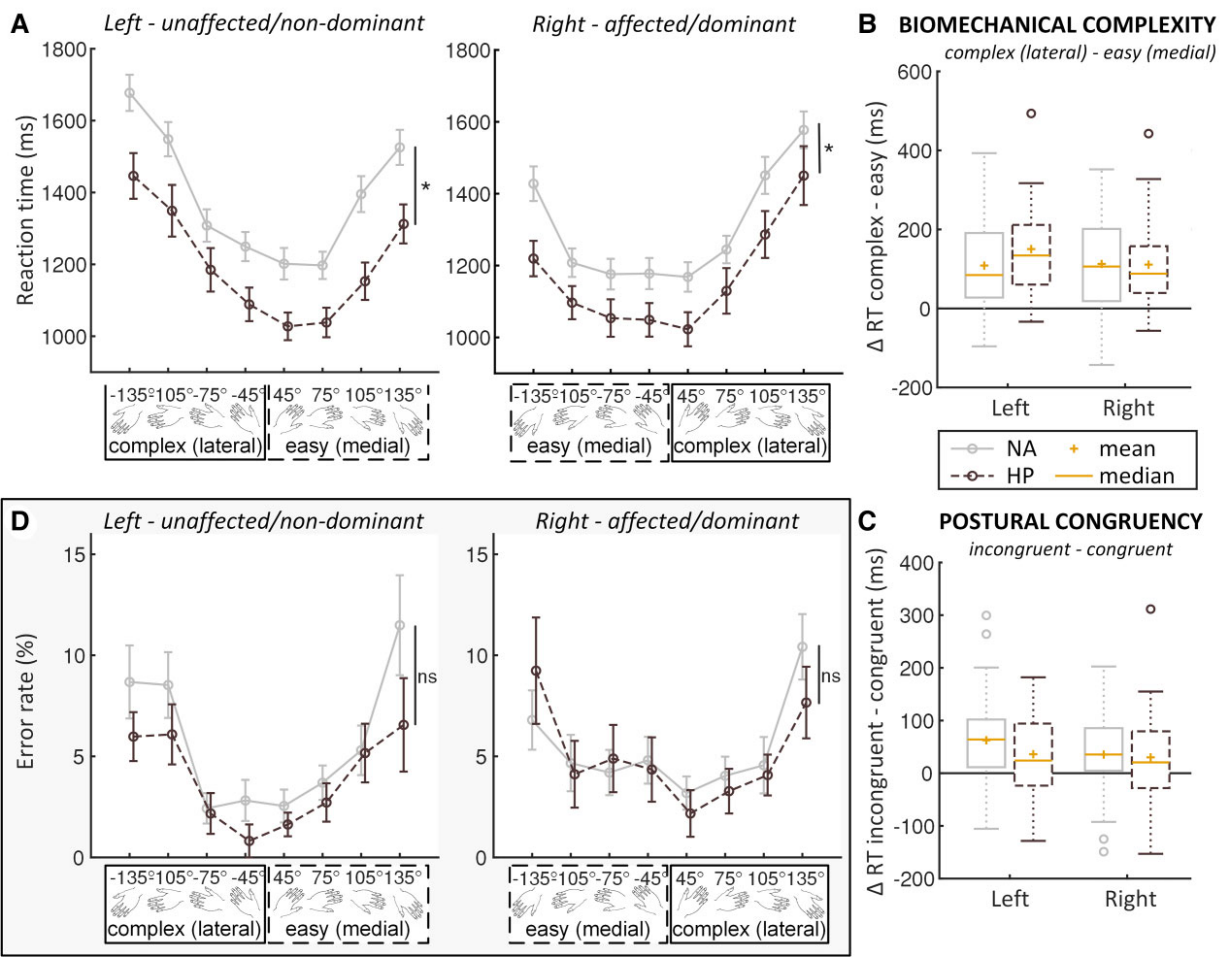
**Behavioural results**. (**A**) Reaction time graphs for left and right hands. As indicated by the asterisks, a 3-factor (GROUP × LATERALITY × BIOMECHANICAL COMPLEXITY) mixed ANOVA revealed a main effect of GROUP [*F*(1,60) = 6.79, *P* < 0.0125, part. *η*^2^ = 0.10]. The *x*-axes of both (**A** and **D**) show the different rotations, collapsed over view (dorsal, palmar), with either a medial/easy or lateral/complex orientation. (**B**) BIOMECHANICAL COMPLEXITY Box plot showing the difference in reaction time between stimuli associated with biomechanically complex and biomechanically easy movement. Positive difference scores indicate significantly slower responses for complex versus easy biomechanical complexities [significant main effect of BIOMECHANICAL COMPLEXITY: *F*(1,60) = 104.60, *P* < 0.001, part. *η*^2^ = 0.64 in the three-way mixed ANOVA of GROUP × LATERALITY × BIOMECHANICAL COMPLEXITY]. (**C**) POSTURAL CONGRUENCY Box plot showing the difference in reaction time between stimuli with a view incongruent versus congruent with participants’ own limb position. Positive difference scores indicate significantly slower responses for incongruent than for congruent postures [significant main effect of POSTURE: *F*(1,60) = 25.15, *P* < 0.001, part. *η*^2^ = 0.30 in the three-way mixed ANOVA of GROUP × LATERALITY × BIOMECHANICAL COMPLEXITY]. In (**B** and **C**) boxes represent the interquartile range (IQR) [from the 25th (Q1) to the 75th (Q3) percentile]; whiskers show the minimum within Q1 − 1.5 × IQR and the maximum within Q3 + 1.5 × IQR; circles are outliers that lie outside Q1/3 ± 1.5 × IQR. (**D**) Error rate graphs showing raw error rate in percentage for left and right hands. As indicated by ns, a 3-factor (GROUP × LATERALITY × BIOMECHANICAL COMPLEXITY) mixed ANOVA revealed that there were no significant group differences in ER [*F*(1,60) = 0.33, *P* = 0.57, part. *η*^2^ = 0.01]. NA = neuralgic amyotrophy; HP = healthy participant; RT = reaction time; ns = not significant; D = delta; ms = milliseconds; *significant difference at *P* < 0.0125.

Overall, NA patients were slower than healthy participants when judging the laterality of hand drawings [significant main effect of GROUP: *F*(1,60) = 6.79, *P* < 0.0125, part. *η*^2^ = 0.10; NA: 1317 ms, healthy: 1153 ms] (Fig. 2A). Both patients and healthy participants were faster with their right hand compared with the left hand (significant main effect of LATERALITY: *F*(1,60) = 12.46, *P* < 0.01, part. *η*^2^ = 0.17; right: 1226 ms, left: 1285 ms). Moreover, both groups were slower for laterally oriented hands (complex), compared with medially oriented hands (easy) [main effect of BIOMECHANICAL COMPLEXITY: *F*(1,60) = 104.60, *P* < 0.001, part. *η*^2^ = 0.64; complex: 1323 ms, easy: 1203 ms] (Fig. 2B). This shows that participants were sensitive to the biomechanical constraints associated with task-related upper limb movements.^19–21,24,25^ Furthermore, both groups were slower for stimuli with a view incongruent with the posture of their own limb, compared with congruent stimuli [main effect of POSTURE: *F*(1,60) = 25.15, *P* < 0.001, part. *η*^2^ = 0.30; incongruent:1279 ms, congruent: 1236 ms] (Fig. 2C). This postural effect confirms that participants incorporated the posture of their own body when performing the task.^21–23^ Neither the effect of BMC, nor the effect of posture differed between groups or per laterality [*F*(1,60) < 1.30, *P* ≥ 0.27, part. *η*^2^ ≤ 0.02].

#### Error rates

Low overall ER showed that participants in both groups performed the task well [mean ± SD, NA patients: 5.5% ± 4.2%; healthy participants 4.4% ± 3.4%; no effect of GROUP: *F*(1,60) = 0.33, *P* = 0.57, part. *η*^2^ = 0.01] (Fig. 2D). ER was similar for left and right hands [no effect of LATERALITY: *F*(1,60) = 0.48, *P* = 0.49, part. *η*^2^ = 0.001], and this did not differ between groups [*F*(1,60) < 3.12, *P* > 0.08, part. *η*^2^ < 0.05]. Neither the BMC, nor the postural congruency of the stimuli influenced ER [*F*(1,60) ≤ 0.50, *P* ≥ 0.48, part. *η*^2^ ≤ 0.008].

### Cerebral activity

#### Shared cerebral activity across groups

**Figure 3:**
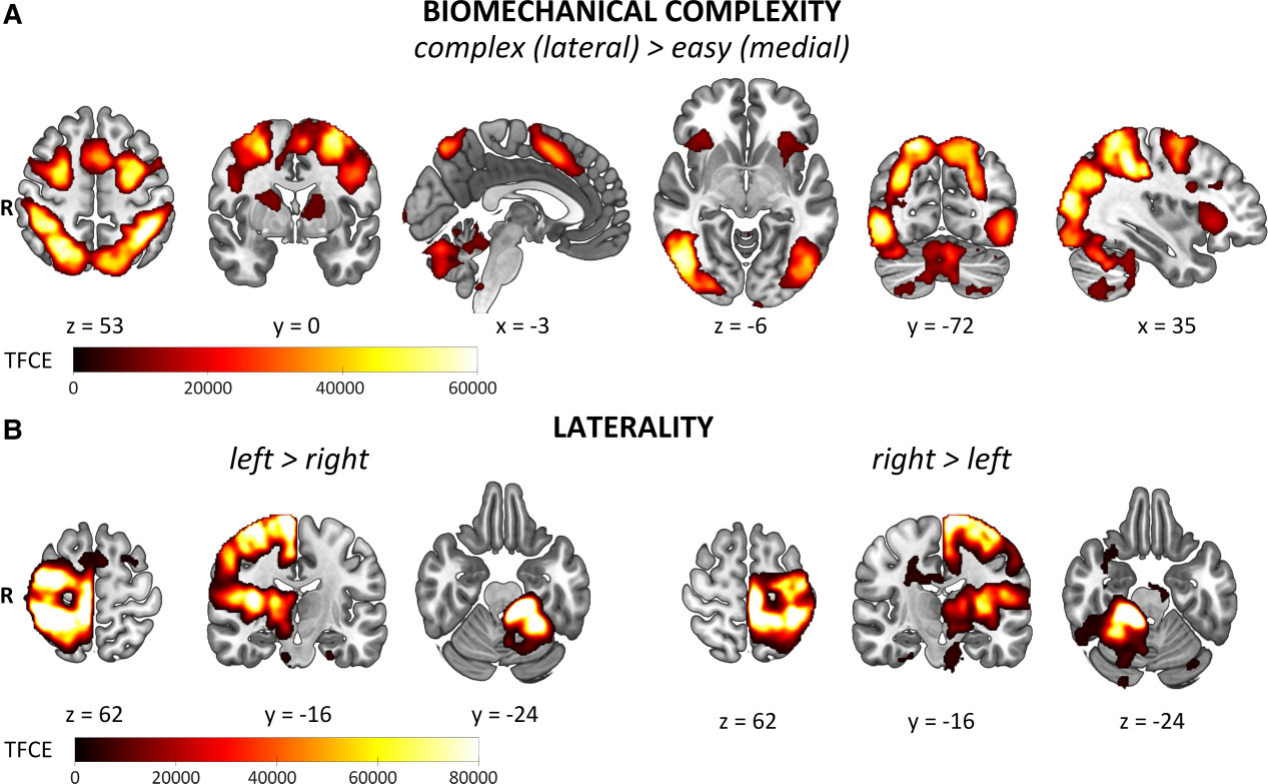
**General task effects**. (**A**) BIOMECHANICAL COMPLEXITY showing TFCE-maps of shared (neuralgic amyotrophy and healthy) activation for biomechanically complex versus easy movements collapsed over laterality. (**B**) LATERALITY shows the shared activity related to factor LATERALITY for left versus right (first three images) and right versus left (last three images) limb movements collapsed over biomechanical complexity. Familywise error corrected, *P* < 0.05; TFCE = threshold-free cluster enhancement; R = right.

The task evoked brain activity related to BMC (complex > easy) in a fronto-parieto-occipital network, including the superior parietal lobule, pre- and postcentral gyrus, superior/middle frontal gyrus, supplementary motor area, inferior/superior lateral occipital cortex, orbitofrontal/insular cortex, thalamus and cerebellum of both hemispheres (Fig. 3A, Table 3). We also found brain activity related to the participants’ own arm posture: if incongruent with the presented stimulus (incongruent > congruent), activity increased in the left precentral gyrus and the right cerebellum. The opposite contrast (congruent > incongruent) was also associated with cerebral activity in the postcentral gyrus, premotor cortex, parietal lobule and superior lateral occipital cortex (see Supplementary Table 2). These findings confirm that both primary somatomotor and visuomotor systems were involved. Finally, we observed brain activity related to the laterality of the stimulus, i.e. activity in a lateralized motor network (contralateral sensorimotor cortex, putamen and thalamus; ipsilateral cerebellum, see Fig. 3B, Table 3). This pattern of activity likely represents the lateralized foot response for each stimulus.

**Table 3:** General task effects table containing information on shared (neuralgic amyotrophy and healthy) activation for the general task effects of BIOMECHANIAL COMPLEXITY (biomechanically complex (lateral) > easy (medial)) and LATERALITY (left > right and right > left)

Anatomical region	*P*-value (FWE-corrected)	Cluster size (voxels)	TFCE (peak voxel)	Stereotactic coordinates (MNI)		
				*x*	*y*	*z*
BIOMECHANICAL COMPLEXITY (shared): complex (lateral) > easy (medial)^a^						
Extensive bilateral activation, most notably: superior parietal lobule/postcentral gyrus/superior lateral occipital cortex	<0.001	14 113	70 249	−44	−42	50
L inferior lateral occipital cortex	<0.001	3957	41 634	−44	−80	0
L middle/superior frontal gyrus/precentral gyrus	<0.001	3782	54 866	−28	−2	60
R middle/superior frontal gyrus	<0.001	1816	50 427	26	−2	54
Bilateral cerebellum, VI/Crus II/Vermis VI	<0.001	661	29 400	−4	−76	−24
R precentral gyrus	<0.001	526	25 567	48	4	26
L insular cortex frontal operculum cortex/orbitofrontal cortex	<0.001	265	19 398	−34	24	2
R frontal operculum cortex/insular cortex/orbitofrontal cortex	<0.001	254	17 704	36	24	0
LATERALITY (shared): left > right						
Extensive activation in right hemisphere, most notably: R postcentral gyrus/primary somatosensory/primary motor cortex/premotor cortex	<0.001	21 480	192 352	8	−38	70
L cerebellum	<0.001	5700	211 684	−16	−32	−24
Brainstem	0.002	209	4810	8	−22	−36
R precentral gyrus	0.020	97	3010	60	10	36
LATERALITY (shared): right > left						
Extensive activation in left hemisphere, most notably: L pre-/postcentral gyrus/inferior/superior parietal lobule	<0.001	23 508	215 520	−4	−32	64
R cerebellum/visual cortex	<0.001	10 430	209 124	10	−38	−24
L cerebellum Crus II/Crus I	0.008	1105	3747	−30	−70	−46
L occipital pole	0.009	552	3719	−16	−98	6
R frontal pole	0.001	496	5144	50	50	4
L superior parietal lobule	0.004	405	4364	−14	−78	38
R temporal pole	0.032	117	2955	34	12	−26
L inferior parietal lobule	0.016	65	3415	−56	−64	10
R hippocampus	0.044	41	2764	36	10	−42
R temporal fusiform cortex/R inferior temporal gyrus	0.044	10	2757	44	−18	−28

As cluster size remained extensive at this threshold, we only report globally on the anatomical regions, and do not provide specific labels and their cluster probability.

L = left; R = right; FWE = familywise error; TFCE = threshold-free cluster enhancement.

^a^ Due to the extent of the cluster for the biomechanical complexity contrast at *P* < 0.05, clusters reported in the table are thresholded at *P* ≤ 0.001 to split the cluster into multiple clusters for reporting.

#### Group differences

**Figure 4:**
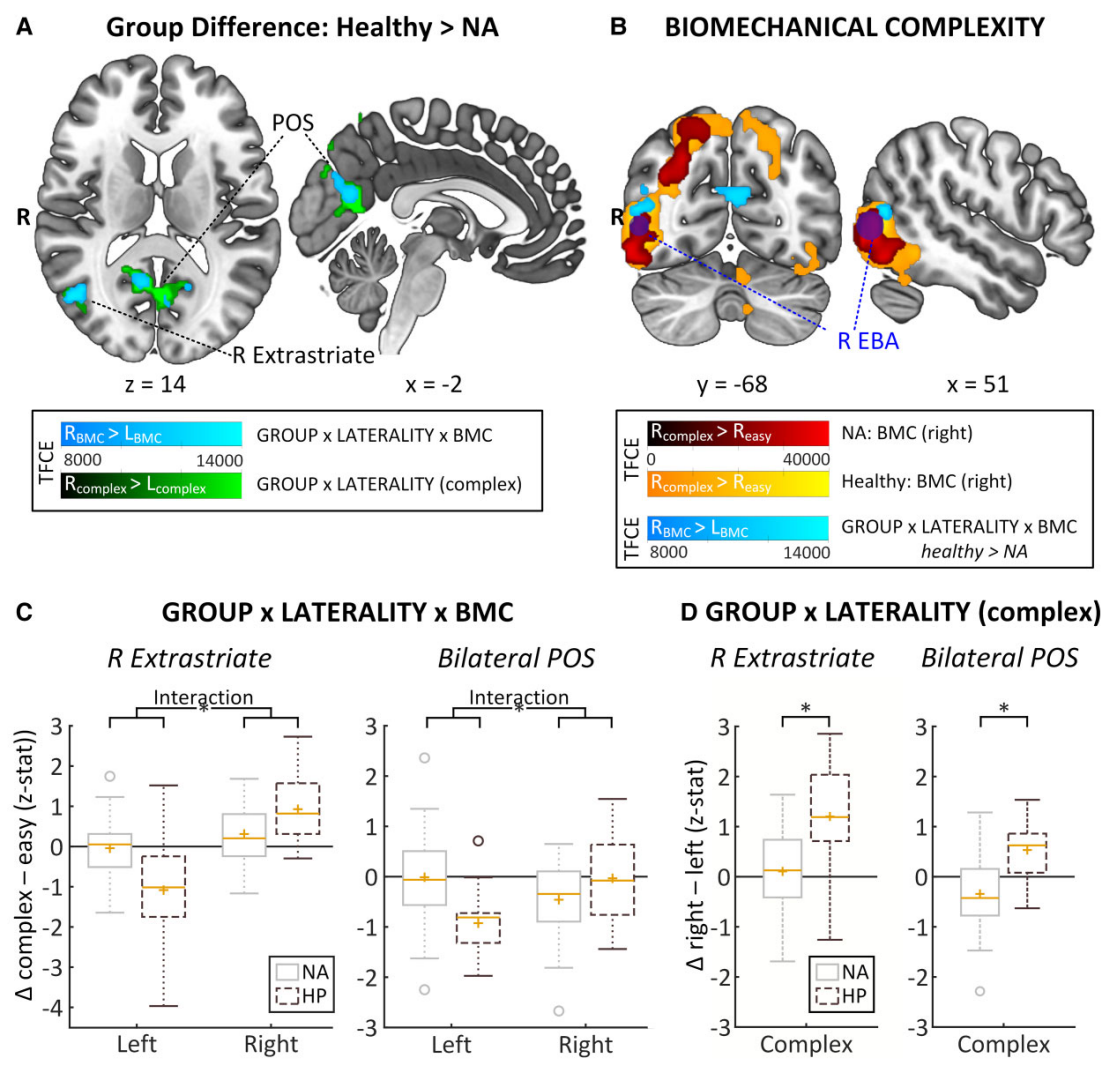
**Main findings**. (**A**) Group difference: Healthy > NA TFCE-maps of where NA patients showed significantly less biomechanical complexity-related activity than healthy participants for two contrasts: the interaction between LATERALITY and BIOMECHANICAL COMPLEXITY (BMC) (in light blue, right_BMC_ > left_BMC,_ where the effect of biomechanical complexity is greater for right than for left hands) and the simpler contrast right_complex_ > left_complex_ (in green) where right is greater for left on complex trials. NA patients had decreased biomechanical complexity-related activity for their right, affected limb in two clusters; the right extrastriate cortex and bilaterally along the parieto-occipital sulcus. NA patients showed decreased activity in those same regions for right versus left complex trials. (**B**) BIOMECHANICAL COMPLEXITY displays how the right extrastriate cluster where healthy > NA (light blue) relates to the right extrastriate body area (dark blue, 8 mm sphere around MNI [50 −73 4], Zimmerman *et al*. [63]) and to the ipsilateral biomechanical complexity-related activation for right hands (i.e. right_complex_ > right_easy_) per group (NA in red, healthy in yellow). (**C**) GROUP × LATERALITY × BMC shows boxplots that display the cluster-specific mean *z*-stat data for the biomechanical complexity effect for left and right hands separately [significant interactions involving group derived from non-parametric TFCE-based permutation testing for the contrast right_BMC_ > left_BMC,_ the interaction term of LATERALITY × BIOMECHANICAL COMPLEXITY: R extrastriate cortex: *P* = 0.011, familywise error corrected, TFCE (peak voxel) = 13 544; bilateral parieto-occipital sulcus: *P* = 0.018, familywise error corrected, TFCE (peak voxel) = 12 679]. (**D**) GROUP × LATERALITY (complex) shows boxplots that display the cluster-specific mean *z*-stat data for right versus left on complex trials [significant difference derived from non-parametric TFCE-based permutation testing for the contrast right_complex_ > left_complex_: R extrastriate cortex: *P* = 0.027, familywise error corrected, TFCE (peak voxel) = 13 167; bilateral parieto-occipital sulcus: *P* = 0.018, familywise error corrected, TFCE (peak voxel) = 17 553]. Boxes represent the interquartile range (IQR) [from the 25th (Q1) to the 75th (Q3) percentile]; whiskers show the minimum within Q1 − 1.5 × IQR and the maximum within Q3 + 1.5 × IQR; yellow lines represent the median, yellow plusses the mean; circles are outliers outside Q1/3 ± 1.5 × IQR. BMC = biomechanical complexity; left/right_BMC_ = biomechanical complexity effect for left/right hands; BMC = biomechanical complexity; EBA = extrastriate body srea; HP = healthy participant; NA = neuralgic amyotrophy; POS = parieto-occipital sulcus R = right; TFCE = threshold-free cluster enhancement; Δ = delta; familywise error corrected at *P* < 0.05. * = significant difference at *P* < 0.05, familywise error corrected see also Table 2 and Supplementary Table 1.

We observed an interaction between GROUP, LATERALITY and BIOMECHANICAL COMPLEXITY. Specifically, when contrasting the effect of BMC of right versus left hands, NA patients showed less brain activity than healthy participants in two clusters: (i) an area in the right extrastriate cortex, located at the junction of the occipital, parietal and temporal lobes, just anterosuperior to the extrastriate body area (EBA), covering parietal area G, posterior (PGp) in the inferior parietal lobule (18%), and part of V5 (12%); and (ii) an area extending bilaterally along the parieto-occipital sulcus (POS), covering parts of Brodmann area (BA) 17 (5% left, 6% right) and 18 (10% left, 8% right) (Fig. 4A, C and D, Table 2). The right extrastriate area falls inside a larger region where brain activity is sensitive to the BMC of right hand stimuli (Fig. 4B, Supplementary Table 1). *Post hoc*, we tested for a difference between right (affected) versus left (unaffected) hand stimuli on trials associated with biomechanically *complex* movements only. This revealed a group difference in the same areas as outlined above: compared with controls, NA patients showed reduced brain activity in right extrastriate cortex (PGp: 21%; V5: 10%) and a cluster along the bilateral POS (BA17: 7% left, 5% right; BA18: 7% left, 7% right), as well as a small cluster in the superior parietal cortex (7A: 5% left, 5% right), specifically during imagery of biomechanically complex movements involving their affected limb (Fig. 4A and D, Table 2). There were no group differences for the main factors LATERALITY, BIOMECHANICAL COMPLEXITY and POSTURE, or for the interaction between LATERALITY and POSTURE.

#### *Post hoc* functional connectivity analysis

Across both groups, the right extrastriate seed was functionally connected to several brain areas that were sensitive to BMC, including cerebellum, premotor and primary motor cortex, frontal gyri and inferior/superior parietal cortex (Supplementary Fig. 1A and Table 3). Across groups, the bilateral POS was functionally connected to regions that together form the default mode network, as well as regions not typically part of the default mode network such as pre- and postcentral gyri (see Supplementary Fig. 1B and Table 3). There were no functional connectivity differences between groups.

### Brain-behaviour-symptom correlations

**Figure 5:**
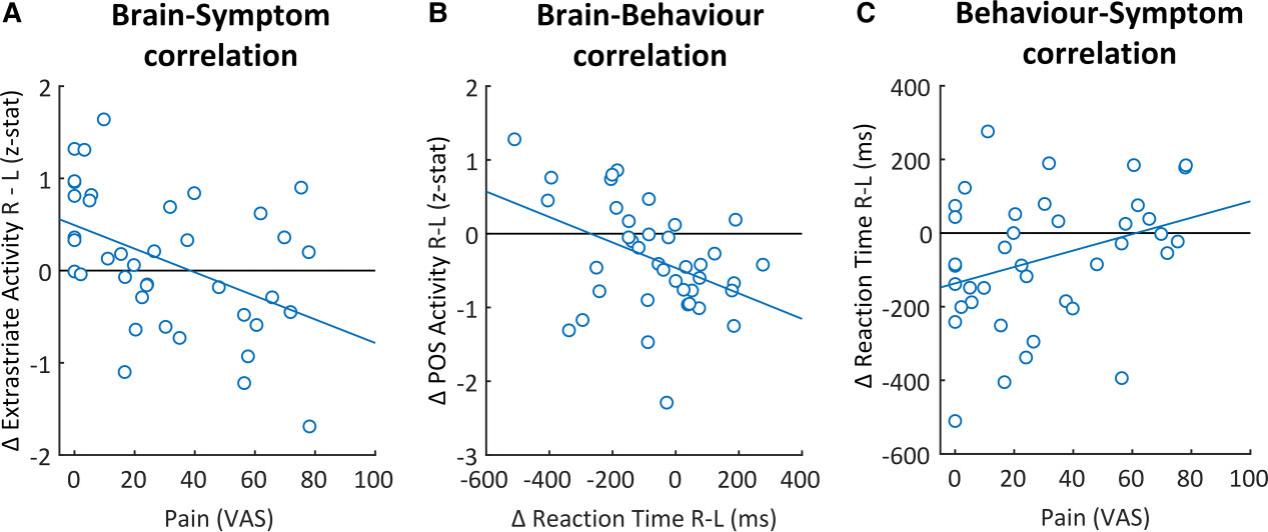
**Brain-behaviour-symptom correlations shows the significant correlations between brain-behaviour-symptom within NA patients**. (**A**) Brain-symptom correlation. Negative correlation (*r* = −0.45, *P* = 0.004) showing that NA patients with more pain had significantly less activity in the right extrastriate cortex when imagining complex movements with the affected limb (right_complex_ > left_complex_). (**B**) Brain-behaviour correlation negative correlation (*r* = −0.42, *P* = 0.008) showing that NA patients with relatively more difficulty with complex movements of the affected limb (i.e. higher Δ reaction time right_complex_ − left_complex_), had less activity along the bilateral parieto-occipital sulcus when imagining those same movements (right_complex_ > left_complex_). (**C**) Behaviour-symptom correlation significant positive correlation (*r* = 0.33, *P* = 0.04) showing that NA patients with more pain had relatively more difficulty with complex trials of the affected limb (i.e. higher Δ reaction time right_complex_ − left_complex_). L = left; ms = milliseconds; POS = parieto-occipital sulcus; R = right; VAS = visual analogue scale; Δ = delta.

NA patients with more persistent pain had significantly less activity in the right extrastriate cortex related to complex movements of the affected limb (*r* = −0.45, *P* = 0.004; Fig. 5A). Other measures of symptom severity showed a similar tendency: patients with less activity in the right extrastriate cortex tended to have lower functional capability of the upper extremity (*r* = −0.30, *P* = 0.066), and a lower relative serratus anterior muscle strength on their affected side (*r* = 0.28, *P* = 0.083). Patients with more pain also had significantly greater difficulty with complex movements of their affected limb (i.e. greater positive difference in RT between affected and unaffected limb, matching the contrast for which patients had decreased cerebral activity in extrastriate cortex and POS) (*r* = 0.33, *P* = 0.04; Fig. 5C) and tended to have higher overall RTs (*r* = 0.29, *P* = 0.07). Moreover, patients with greater difficulty with complex movements of their affected limb had significantly less activity along the POS (*r* = −0.42, *P* = 0.008; Fig. 5B), but not in the right extrastriate cortex (*r* = −0.08, *P* = 0.62), when imagining those same movements. Behaviour did not correlate with brain activity in either region in healthy participants (POS: *r* = −0.12, *P* = 0.56; right extrastriate: *r* = −0.02, *P* = 0.93). Symptom severity did not correlate significantly with brain activity in the POS (*r* < ±0.17, *P* > 0.31) or with overall RT (*r* < ±0.29, *P* ≥ 0.07).

## Discussion

In this fMRI study, we show that patients with a lateralized peripheral nervous system disorder (i.e. NA of the brachial plexus, affecting the right upper extremity) have altered cerebral and behavioural responses during hand laterality judgement. Specifically, compared with healthy participants, NA patients were overall slower, and had decreased cerebral activity when mentally rotating their affected limb in two brain regions: right extrastriate cortex and bilateral POS. This indicates that NA patients have altered sensorimotor representations of their affected upper extremity. It also suggests that this maladaptive cerebral neuroplasticity arises from visuomotor rather than primary somatomotor regions. Exploratory analyses revealed that patients with greater symptom severity were relatively slower and had decreased cerebral activity when imagining movements with their affected limb, which may suggest a link between altered sensorimotor representations and clinical outcome in NA.

### Imagery-related effects in right extrastriate cortex

NA patients had decreased activity in right extrastriate cortex when imagining complex movements with their affected (right) limb. In both healthy participants and NA patients, motor imagery of biomechanically complex movements with the right limb evoked robust activity in the ipsilateral (right) extrastriate cortex, as well as other areas along the right ventral and dorsal visual streams. Similar findings have been observed in other populations.^30^ The decreased activity in NA patients in right extrastriate cortex was observed at the junction between occipital, parietal and temporal lobes, with partial overlap with V5 and inferior parietal area PGp, just anterior and superior to the EBA. The EBA is defined by its sensitivity to body parts,^59,60^ particularly when those percepts need to be translated in motor plans,^61–65^ as during the hand laterality task.^30,31,66^ These functional properties extend to other extrastriate areas, including V5 and parietal areas in the dorsal visual stream.^62,67–69^ The receptors fingerprint of area PGp, another element of our extrastriate cluster, is similar to the fingerprint of extrastriate cortex, and it is similarly connected with occipital and parietal areas.^70–72^ Accordingly, we found that the right extrastriate cluster was functionally connected to areas in dorsal and ventral visual streams, as well as to posterior parietal and premotor areas. The EBA works together with posterior parietal and premotor cortex to form a sensorimotor representation of the own upper limb through integration of visual and proprioceptive information.^63,65,73–75^ More precisely, it has been argued that the EBA is an important interface between visual perception and action,^61^ calculating a predicted goal posture during motor planning.^63–65^ The decrease in extrastriate imagery-related activity as persistent pain increased thus suggests that persistent pain related to motor dysfunction reduced patients’ reliance on goal posture predictions when solving the motor imagery task. In agreement with our observation, the EBA has been shown to be involved in anticipation and observation of painful movements, and first-person perspective pain observation.^76,77^ These considerations qualify previous reports on the consequences of persistent pain in other peripheral disorders,^78–82^ suggesting that altered visuomotor processing may underly the effects of persistent pain on patients’ ability to imagine biomechanically complex movements of the affected limb. This interpretation is in line with the well-known, strong relation between altered scapular biomechanics and persistent pain in NA.^12,13,15^

### Imagery-related effects in bilateral parieto-occipital sulcus

NA patients also had decreased activity along the POS, covering parts of the ventral posterior cingulate cortex and the precuneus. This area forms a major hub in various brain circuits.^83–85^ In rest and task-unfocused mindsets, it is involved in internal modes of cognition as part of the default mode network.^84–88^ In task-focused mindsets, this higher-order visual region contributes to a multisensory representation of the spatial location of the own body through integration of self-relevant information.^74,83,84,88–91^ Patients with more behavioural difficulty in performing the task with their affected limb (as indexed by RTs) had less activity along the POS. Our findings could potentially be interpreted as reflecting deactivation of the default mode network, as it is known to deactivate with increasing task difficulty.^87^ However, although the cluster in the POS was functionally connected to other regions that are part of the default mode network, there were no group differences in functional coupling between those regions. Moreover, the POS cluster was also functionally connected to regions that are not part of the default mode network, such as somatosensory and somatomotor areas.^91^ These connections fit with the region’s task-focused functions. The decreased activity along the POS thus likely reflects altered processing and integration of self-relevant visuomotor information in NA patients.

### Behavioural task effects

While the cerebral alterations in NA patients were specific to the affected upper extremity, patients were slower than healthy participants with both their affected and unaffected extremity. This deviates from our previous behavioural study, where we found decreased accuracy specific to the affected limb in an independent sample of NA patients.^18^ Interestingly, both unilateral and bilateral deficits in both RT and accuracy have been reported in other asymmetric upper extremity disorders.^28,33,80,82,92–94^ These differing results may stem from differences in patient populations and experimental settings. NA patients in the current study were tested at an earlier disease stage (median/mean of 8/17 months after onset) than our previous sample (16/61 months).^18^ Furthermore, here patients performed the task in a supine position while lying in the MRI-scanner, whereas the patients in our previous behavioural study were sitting upright in a more natural environment.^18^ The effect of the MRI-scanner environment on performance may have differed between patients and healthy participants, which could explain why patients were slower, but did not show decreased accuracy compared with the healthy participants. Likewise, the overall slowing of responses could be a generic consequence of residual NA symptoms like fatigue,^12,13,15^ which may have been exacerbated by the demanding MRI-scanner environment.^95^ Alternatively, the bilateral behavioural impairment may reflect an increased reliance on sensorimotor representations of the unaffected extremity, subsequently slowing responses for both extremities.^94^ Importantly, the *cerebral* alterations were specific to the affected limb, which might reflect the ability of fMRI to capture differences in cerebral processes between conditions, even when behavioural performance (e.g. RT) stays constant.^96–99^ Our finding that increased activity along the POS correlated with faster RTs (for the affected limb) suggests that this region is relevant for task performance.^96,99^ Furthermore, the negative correlation between task-related activity in the extrastriate cortex and residual NA symptoms (persistent pain related to motor dysfunction) suggests that this brain region is also relevant for NA. Follow-up longitudinal studies might be able to characterize the relative dynamics of cerebral and behavioural alterations in NA.

### Interpretational issues

There has been some debate on the exact processes underlying hand laterality. It has been mainly assumed that this task involves implicit motor imagery; whereby participants imagine moving their hand to match the stimulus hand.^20,24,26,41,92^ Recently, it has been proposed that this task does not involve motor imagery, but rather relies on visual strategies^100,101^ and multisensory integration, whereby multisensory binding of the proprioceptive representation of the own hand and the visual representation of the hand stimulus are used to determine hand laterality.^102^ Both motor imagery and multisensory integration involve sensorimotor representations of the upper extremity, which means that hand laterality judgement can be utilized to study these representations in both scenarios. Importantly, we included manipulations of postural congruency and BMC in our design to provide empirical evidence that participants used their own body as a reference during the task and, thus, that they did not identify hand laterality through purely visual strategies. The fact that both behavioural and cerebral responses were sensitive to these experimental manipulations confirms that participants employed an embodied strategy, which is corroborated by a large body of evidence.^19,21–23,29^

Other features of our experimental design exclude that our findings are a generic consequence of disease-related factors, like peripheral changes and generic effects of symptoms as fatigue. For instance, participants responded flexing their toes, an experimental choice guided by the fact that the patients’ lower limbs were not affected by the disorder. Likewise, the selection of patients with unilateral symptoms, in combination with the inclusion of the factor laterality in our main analyses, provided a within-subject control, which allowed us to compare the affected and unaffected limb. The specificity of the cerebral effects to the affected upper extremity excludes that these effects stem from generic group differences in experienced task difficulty due to factors like fatigue.

### Conclusion and clinical implications

Our findings suggest that maladaptive cerebral plasticity plays a role in residual motor dysfunction and subsequent persistent pain in NA. Our data localize cerebral changes in NA to visuomotor brain regions involved in sensorimotor integration, i.e. the right extrastriate cortex (close to the EBA) and the bilateral POS. This may have important implications for treatment of NA, and possibly for other peripheral nerve disorders. For example, coordinative motor training with online visual feedback of the shoulder is one of the most effective treatments for residual complaints in NA.^12,17^ Likewise, visuomotor approaches targeting sensorimotor integration in other neural disorders include augmented (visual) feedback, action observation and graded motor imagery.^103–105^ Our findings suggest that a focus on visual feedback may further improve these treatments, especially for patients with motor dysfunction who experience persistent pain.

## Supplementary Material

fcac034_Supplementary_Data

## Abbreviations

BA =: Brodmann area
BET =: brain extraction tool
BMC =: biomechanical complexity
BOLD =: blood-oxygen-level dependent
DASH =: disabilities of the arm, shoulder and hand
EBA =: extrastriate body area
ER =: error rate
FEAT =: fMRI expert analysis tool
FLIRT =: FMRIB’s linear image registration tool
fMRI =: functional magnetic resonance imaging
FNIRT =: FMRIB’s non-linear image registration tool
FOV =: field of view
FWE =: family wise error
FWHM =: full width at half maximum
GLM =: general linear model
HRF =: hemodynamic response function
ICA-AROMA =: independent component analysis for automatic removal of motion artefacts
L =: left
MB6 =: multiband 6
MPRAGE =: magnetization prepared rapid gradient echo
NA =: neuralgic amyotrophy
PGp =: parietal area G, posterior
POS =: parieto-occipital sulcus
R =: right
RCT =: randomized controlled trial
RT =: reaction time
TE =: echo time
TFCE =: threshold-free cluster enhancement
TI =: inversion time
TR =: repetition time
VAS =: visual analogue scale
