# Supplementary material for: Visuomotor processing is altered after peripheral nerve damage in neuralgic amyotrophy
Source: Brain Commun. 2022 Feb 16;4(1):fcac034. doi: 10.1093/braincomms/fcac034 (PMC8882006; doi:10.1093/braincomms/fcac034)
Supplement: fcac034_Supplementary_Data [file fcac034_supplementary_data.pdf]

Supplementary Materials

Detailed description of fMRI preprocessing steps

First, we converted all functional and structural DICOM files to Niftii files using dcm2nii.[1] Non-brain structures such as skull were removed from the structural image using the Brain Extraction Tool (BET2).[2] If BET brain extraction did not lead to a satisfactory removal of skull and a subsequent satisfactory registration, excessive skull was removed manually (n =1). We calculated the framewise displacement for the functional scans with fsl_motion_outliers and excluded participants with a mean framewise displacement > 0.5mm. The first five volumes of the functional data were removed to allow signal equilibration of the scanner. Images were realigned to correct for motion with fMRI Expert Analysis Tool version 6.00 (FEAT).[3] FEAT additionally applied smoothing (Full Width at Half Maximum (FWHM) = 3 mm) and grand mean scaling, to normalize the mean of each voxel, and removed non-brain structures from the functional images. Functional images were registered to both the subject's structural image and to standard MNI152 image space using FMRIB's Linear Image Registration Tool (FLIRT).[4, 5] Registration from high resolution structural to standard space was then further refined using nonlinear registration (FNIRT).[6, 7] Motion-related noise was removed from our data using ICA-AROMA,[8] set to generate 100 independent components per participant, which were manually inspected, heeding the spatial pattern, time series and frequency spectrum,[9] to ensure correct classification as noise or signal and were reclassified if needed. Non-aggressive denoising was then applied using ICA-AROMA with the reclassified components. Next, we performed nuisance regression on the denoised images using fslmaths and fslglm. This nuisance regression included regressors of white matter and cerebrospinal fluid, as well as 24 motion parameters. White matter and cerebrospinal fluid masks were created through tissue-type segmentation with FMRIB's Automated Segmentation Tool (FAST),[10] eroded with fslmaths and registered to functional space using FLIRT. Average time series of these masks were subtracted using fslmeants and added as regressors. The motion regressors consisted of six primary realignment parameters, the history of these realignment parameters, and the squared parameters.[11] After nuisance regression, we temporally high pass filtered the data at 0.01 Hz and applied additional smoothing with a 5.2 mm FWHM Gaussian kernel, amounting to a final smoothing of 6mm FWHM.

Supplementary Table 1 Biomechanical complexity-related activity for right hands table containing information on activation for the effect of BIOMECHANICAL COMPLEXITY for right hands (i.e. $right_{complex} > right_{easy}$). Due to the extent of the clusters, we only report the anatomical regions globally and do not provide specific labels and their cluster probability. (see also Fig. 4B) L = left; R = right; FWE = familywise error; TFCE = threshold-free cluster enhancement

**Anatomical region**	**p-value (FWE-corrected)**	**Cluster size (voxels)**	**TFCE (peak voxel)**	**Stereotactic coordinates (MNI)**		
				**x**	**y**	**z**
**BIOMECHANICAL COMPLEXITY (right): $right_{complex} > right_{easy}$**						
**NA**						
R parietal lobule / visual cortex	< 0.001	3006	45966	34	-84	14
L visual cortex V1/V2/V3V/V4	0.004	285	26204	-28	-92	-16
L intraparietal sulcus / superior parietal lobule	0.015	206	22378	-32	-48	44
R middle/superior frontal gyrus	0.025	65	20813	-28	-2	60
R intraparietal sulcus / superior parietal lobule / primary somatosensory cortex	0.045	27	19291	34	-42	48
R middle/superior frontal gyrus / precentral gyrus	0.046	12	19184	28	-2	52
L intraparietal sulcus	0.048	10	19090	-28	-62	40
L precentral gyrus / middle frontal gyrus	0.049	4	18991	-44	2	34
**healthy**						
Bilateral parietal lobule / precuneus/ visual cortex / temporal gyri	< 0.001	7628	52233	32	-86	22
L visual cortex V1/V2	< 0.001	1797	19079	-16	-100	-2
Cerebellum L VI, Vermis VI	0.011	210	11277	-6	-68	-24
L cerebellum VIII	0.045	17	8434	-32	-44	-54
L cerebellum X	0.049	2	8288	-24	-32	-42
L middle frontal gyrus	0.050	2	8253	-32	-2	60

Supplementary Table 2 Posture-related activity table containing information on shared (neuralgic amyotrophy and healthy) activation for the effect of POSTURE (incongruent > congruent, congruent > incongruent) L = left; R = right; FWE = familywise error; TFCE = threshold free cluster enhancement.

Anatomical region	Cluster probability (%) Juelich Histological atlas		p-value (FWE corrected)	Cluster size (voxels)	TFCE (peak voxel)	Stereotactic coordinates (MNI)		
						x	y	z
**POSTURE (shared): incongruent > congruent**								
L precentral gyrus			0.046	3	10702	-28	-10	46
	L premotor cortex BA6	19%						
R cerebellum Crus I		79%	0.046	2	10685	36	-70	-24
**POSTURE (shared): congruent > incongruent**								
bilateral visual cortex			0.002	1579	17702	-6	-68	2
	L V1 BA17	23%						
	R V1 BA17	18%						
	L V2 BA18	17%						
	R V2 BA18	14%						
R postcentral gyrus / precentral gyrus			0.007	844	13903	48	-26	46
	R primary somatosensory cortex							
	BA1	24%						
	BA3b	19%						
	BA2	16%						
	R premotor cortex BA6	21%						
	R primary motor cortex							
	BA4	17%						
	BA4p	10%						
L precentral gyrus / postcentral gyrus			0.012	279	12984	-28	-32	68
	L primary somatosensory cortex							
	BA3b	29%						
	BA1	18%						
	L primary motor cortex							
	BA4p	25%						
	BA4a	25%						
	L premotor cortex BA6	20%						
R visual cortex			0.024	138	11616	14	-82	28
	R V2 BA18	17%						
R superior parietal lobule			0.023	88	11695	16	-78	46
	R superior parietal lobule 7P	16%						
R superior/inferior parietal lobule			0.049	2	10228	44	-42	60
	R superior parietal lobule 7PC	17%						
	R inferior parietal lobule 7Pm	14%						

Our cerebral findings with regard to the postural congruency-effect were somewhat unexpected based on traditional reports (i.e. more activity for congruent > incongruent rather than for incongruent > congruent as reported by de Lange et al.[12] and Helmich et al.[13]. This can however be explained by the fact that the manipulation of limb-posture differed between studies. Moreover, more recent work has reported activity for congruent versus incongruent postures similar to our data.[14] Importantly, the behavioural advantage for congruent limb-postures and the involvement of parietal cortex, post- and precentral gyri, confirm the embodied nature of the task.

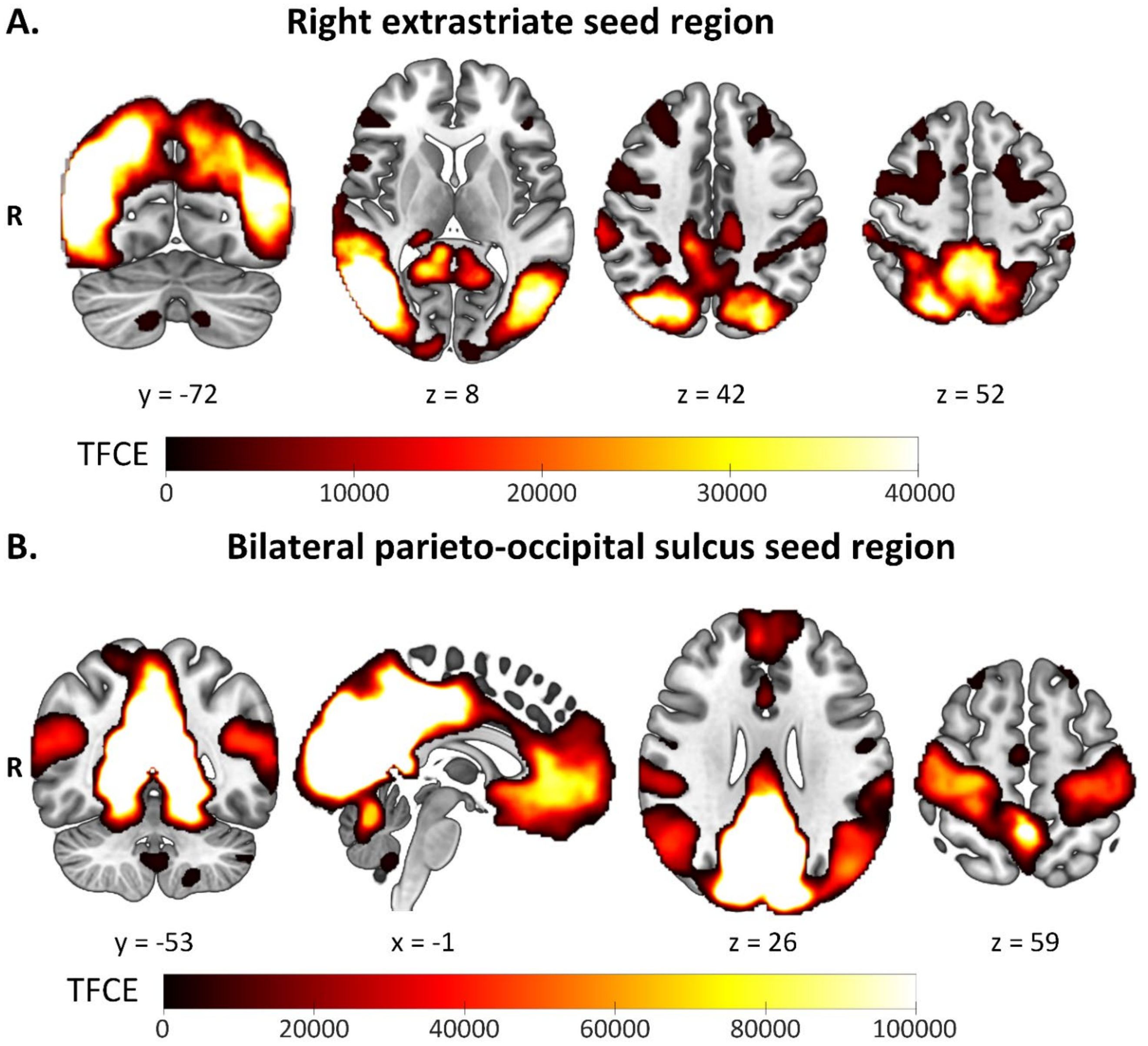

Supplementary Figure 1 Seed region analyses for extrastriate cortex and parieto-occipital sulcus TFCE-maps of shared (neuralgic amyotrophy and healthy) functional connectivity with (**A.**) the right extrastriate cortex seed region and (**B.**) the bilateral parieto-occipital seed region. Seed-regions were derived from the interaction between LATERALITY x BIOMECHANICAL COMPLEXITY x GROUP (i.e. $Right_{BMC} > Left_{BMC}$; healthy > neuralgic amyotrophy). Familywise error corrected, $p < 0.05$; $Left/Right_{BMC}$ = biomechanical complexity effect for left/right hands; TFCE = threshold free cluster enhancement; R = right

Supplementary Table 3 seed-region analyses table containing information on shared (neuralgic amyotrophy and healthy) functional connectivity of the right extrastriate and the bilateral parieto-occipital sulcus seed regions, derived from the interaction between LATERALITY x BIOMECHANICAL COMPLEXITY x GROUP (i.e. $Right_{BMC} > Left_{BMC}$ – healthy > neuralgic amyotrophy). Due to the extent of the clusters, we only report the anatomical regions globally and do not provide specific labels and their cluster probability. See Supplementary Figure 1 for the TFCE-maps. BMC = biomechanical complexity; L = left; R = right; FWE = familywise error; TFCE = threshold free cluster enhancement

Anatomical region	p-value (FWE corrected)	Cluster size (voxels)	TFCE (peak voxel)	Stereotactic coordinates (MNI)		
				x	y	z
**Right extrastriate seed region connectivity (shared)**						
Extensive bilateral connectivity, most notably: superior/inferior parietal lobule / premotor cortex / visual cortex	< 0.001	55937	863761	46	-64	18
L middle/superior frontal gyrus/ frontal pole	0.012	327	2036	-28	32	44
R frontal pole / inferior frontal gyrus, pars triangularis	0.020	246	1715	54	38	8
L inferior frontal gyrus, pars triangularis	0.022	246	1672	-42	34	10
L inferior frontal gyrus, pars opercularis	0.027	199	1583	-42	8	24
R frontal pole / paracingulate gyrus / frontal medial cortex	0.029	162	1555	2	52	-8
R cerebellum	0.007	133	2284	12	-76	-46
L cerebellum	0.012	78	2019	-12	-74	-44
L cerebellum	0.002	58	2857	-10	-46	-48
bilateral anterior cingulate gyrus	0.036	51	1483	0	6	30
L frontal medial cortex / paracingulate gyrus	0.043	22	1406	-10	34	-14
R paracingulate gyrus / frontal medial cortex	0.043	21	1405	12	34	-10
R cerebellum	0.037	8	1477	12	-48	-52
L superior temporal gyrus	0.047	5	1371	-58	-6	-6
**Bilateral parieto-occipital sulcus seed region connectivity (shared)**						
Extensive bilateral connectivity, most notably: precuneus / cingulate gyrus/ frontal pole/ occipital pole/ temporal pole / superior lateral occipital cortex/ pre-/postcentral gyrus / lingual gyrus	< 0.001	77678	1377830	6	-64	18
R superior frontal gyrus / frontal pole / middle frontal gyrus	< 0.001	760	5802	26	34	48
Cerebellum	< 0.001	162	4792	-4	-52	-44
L cerebellum	0.010	97	2749	-22	-52	-52
L orbitofrontal cortex / frontal pole	0.014	61	2489	-32	34	-14
R cerebellum	0.006	49	3059	16	-86	-40

References

1. Li X, Morgan PS, Ashburner J, Smith J, Rorden C. The first step for neuroimaging data analysis: DICOM to NIfTI conversion. *J Neurosci Methods*. May 1 2016;264:47-56. doi:10.1016/j.jneumeth.2016.03.001
2. Smith SM. Fast robust automated brain extraction. *Hum Brain Mapp*. Nov 2002;17(3):143-55. doi:10.1002/hbm.10062
3. Woolrich MW, Ripley BD, Brady M, Smith SM. Temporal autocorrelation in univariate linear modeling of FMRI data. *Neuroimage*. Dec 2001;14(6):1370-86. doi:10.1006/nimg.2001.0931
4. Jenkinson M, Bannister P, Brady M, Smith S. Improved optimization for the robust and accurate linear registration and motion correction of brain images. *Neuroimage*. Oct 2002;17(2):825-41. doi:10.1016/s1053-8119(02)91132-8
5. Jenkinson M, Smith S. A global optimisation method for robust affine registration of brain images. *Med Image Anal*. Jun 2001;5(2):143-56. doi:10.1016/s1361-8415(01)00036-6
6. Andersson JLR, Jenkinson M, Smith S. *Non-linear optimisation FMRIB Technial Report TR07JA1*. 2007.
7. Andersson JLR, Jenkinson M, Smith S. *Non-linear registration aka Spatial normalisation FMRIB Technial Report TR07JA2*. 2007.
8. Pruim RHR, Mennes M, van Rooij D, Llera A, Buitelaar JK, Beckmann CF. ICA-AROMA: A robust ICA-based strategy for removing motion artifacts from fMRI data. *Neuroimage*. May 15 2015;112:267-277. doi:10.1016/j.neuroimage.2015.02.064
9. Griffanti L, Douaud G, Bijsterbosch J, et al. Hand classification of fMRI ICA noise components. *Neuroimage*. Jul 1 2017;154:188-205. doi:10.1016/j.neuroimage.2016.12.036
10. Zhang Y, Brady M, Smith S. Segmentation of brain MR images through a hidden Markov random field model and the expectation-maximization algorithm. *IEEE Trans Med Imaging*. Jan 2001;20(1):45-57. doi:10.1109/42.906424
11. Satterthwaite TD, Elliott MA, Gerraty RT, et al. An improved framework for confound regression and filtering for control of motion artifact in the preprocessing of resting-state functional connectivity data. *Neuroimage*. Jan 1 2013;64:240-56. doi:10.1016/j.neuroimage.2012.08.052
12. de Lange FP, Helmich RC, Toni I. Posture influences motor imagery: an fMRI study. *NeuroImage*. Nov 01 2006;33(2):609-17. doi:10.1016/j.neuroimage.2006.07.017
13. Helmich RC, de Lange FP, Bloem BR, Toni I. Cerebral compensation during motor imagery in Parkinson's disease. *Neuropsychologia*. Jun 11 2007;45(10):2201-15. doi:10.1016/j.neuropsychologia.2007.02.024
14. Qu F, Wang J, Zhong Y, Ye H. Postural Effects on the Mental Rotation of Body-Related Pictures: An fMRI Study. *Front Psychol*. 2018;9:720. doi:10.3389/fpsyg.2018.00720
